# Isoflurane-Induced Burst Suppression Increases Intrinsic Functional Connectivity of the Monkey Brain

**DOI:** 10.3389/fnins.2019.00296

**Published:** 2019-04-11

**Authors:** Zhao Zhang, Dan-Chao Cai, Zhiwei Wang, Kristina Zeljic, Zheng Wang, Yingwei Wang

**Affiliations:** ^1^Department of Anesthesiology, Huashan Hospital, Fudan University, Shanghai, China; ^2^Institute of Neuroscience, State Key Laboratory of Neuroscience, Key Laboratory of Primate Neurobiology, CAS Center for Excellence in Brain Science and Intelligence Technology, Shanghai Institutes for Biological Sciences, Chinese Academy of Sciences, Shanghai, China; ^3^University of Chinese Academy of Sciences, Beijing, China; ^4^Kunming Institute of Zoology, Chinese Academy of Sciences, Kunming, China

**Keywords:** burst suppression, simultaneous EEG-MRI, functional connectivity, default mode network, thalamocortical connectivity, nonhuman primate

## Abstract

Animal functional magnetic resonance imaging (fMRI) has provided key insights into the physiological mechanisms underlying healthy and diseased brain states. In non-human primates, resting-state fMRI studies are commonly conducted under isoflurane anesthesia, where anesthetic concentration is used to roughly infer anesthesia depth. However, within the recommended isoflurane concentration range (1.00–1.50%), the brain state can switch from moderate anesthesia characterized by stable slow wave (SW) electroencephalogram (EEG) signals to deep anesthesia characterized by burst suppression (BS), which is electrophysiologically distinct from the resting state. To confirm the occurrence rate of BS activity in common setting of animal fMRI study, we conducted simultaneous resting-state EEG and fMRI experiments on 16 monkeys anesthetized using 0.80–1.30% isoflurane, and detected BS activity in two of them. Datasets either featured with BS or SW activity from these two monkeys were analyzed to investigate the intrinsic functional connectivity (FC) patterns during BS. In datasets with BS activity, we observed robust coupling between the BS pattern (the binary alternation between burst and suppression activity in EEG signal) and filtered BOLD signals in most brain areas, which was associated with a non-specific enhancement in whole brain connectivity. After eliminating the BS coupling effect by regressing out the BS pattern, we detected an overall increase in FC with a few decreased connectivity compared to datasets with SW activity. These affected connections were preferentially distributed within orbitofrontal cortex, between orbitofrontal and prefrontal/cingulate/occipital cortex, and between temporal and parietal cortex. Persistence of the default mode network and recovery of thalamocortical connections were also detected under deep anesthesia with BS activity. Taken together, the observed spatially specific alterations in BS activity induced by isoflurane not only highlight the necessity of EEG monitoring and careful data preprocessing in fMRI studies on anesthetized animals, but also advance our understanding of the underlying multi-phased mechanisms of anesthesia.

## Introduction

Functional magnetic resonance imaging (fMRI) has become a commonly used noninvasive technique for brain activity research in both animals and humans. Resting-state fMRI (rs-fMRI) measures functional connectivity (FC) between different brain regions in the non-stimulus state and is a valuable technique for exploring complex brain networks in different brain states or disease models (Greicius, 2008; Van Den Heuvel and Hulshoff Pol, 2010). Anesthesia is an effective method to prevent head motion and physiological stress when collecting blood oxygenation level dependent (BOLD) fMRI signals, particularly in animal studies. On the other hand, it is also a critical confounding factor because anesthetic selection and dosage can affect or even inverse the intrinsic BOLD fluctuations readout (Hutchison and Everling, 2012; Grandjean et al., 2014; Lv et al., 2016; Paasonen et al., 2017, 2018; Wu et al., 2017; Bukhari et al., 2018). For instance, isoflurane, the most prevalent inhaled anesthetic for monkey fMRI study, has a significant, concentration-dependent influence on FC networks (Vincent et al., 2007; Nallasamy and Tsao, 2011; Grandjean et al., 2014; Hutchison et al., 2014; Bukhari et al., 2018). A limited range of isoflurane concentration (1.00–1.50%) is therefore recommended to obtain stable FC in anesthetized nonhuman primate resting-state experiments (Hutchison et al., 2014).

However, monkey fMRI studies with simultaneously recorded electroencephalogram (EEG) signals have revealed that relatively deep anesthesia characterized by burst suppression (BS) occasionally occurs at the recommended dosage of isoflurane (end-tidal concentration 1.25–1.50%) (Vincent et al., 2007). BS is a stereotypic electrical activity pattern in the brain that presents as continuous alternation between two states, high-voltage waves (burst) and isoelectric epoch (suppression), and is fundamentally different from the slow wave (SW) activity most commonly observed in the resting state (Steriade et al., 1994). Electrophysiological studies have demonstrated wide synchronization and hyper-excitation across the whole brain during BS activity (Steriade et al., 1994). A robust coupling effect between BS pattern (the binary alternation between burst and suppression activity in EEG signal) and BOLD fluctuations has been revealed in sevoflurane-anesthetized humans (Golkowski et al., 2017). Enhanced BOLD connectivity in the somatosensory network has also been reported in rats during isoflurane-induced BS activity (Liu et al., 2011). Further investigation of the same network showed that connectivity became less spatially specific as anesthesia depth increased to BS status, evidencing functional reorganization of the brain (Liu et al., 2013). These findings indicate multiphasic progression from light to deep anesthesia, suggesting a non-linear relationship between brain activity strength and anesthesia depth (Amzica, 2015).

Here, we aim to explore the signature of the whole-brain functional network connectivity during the isoflurane-induced BS activity in adult macaque monkeys. We are also concerned with the influence of preprocessing strategy on the spatially non-specific increase in FC reported by Liu et al. (2013). We are particularly interested in alterations of the default mode network (DMN) and thalamocortical connections, both of which are sensitive to anesthesia (Alkire et al., 1999; Fiset et al., 1999; Vogt and Laureys, 2005). Despite observed decreases in both DMN (Greicius et al., 2008; Huang et al., 2014) and thalamocortical connectivity (White and Alkire, 2003; Huang et al., 2014) under anesthesia compared to the awake state in humans, it is unknown whether or not these two networks are present during the EEG-defined BS activity in nonhuman primates.

## Materials and Methods

### Participants

All experimental procedures for nonhuman primate research in this study were approved by the Institutional Animal Care and Use Committee at the Institute of Neuroscience and the Biomedical Research Ethics Committee, Shanghai Institutes for Biological Sciences, Chinese Academy of Sciences, and conformed to National Institutes of Health guidelines for the humane care and use of laboratory animals.

The data included in this work were selected from a series of simultaneous resting-state EEG and fMRI experiments of 16 monkeys (*Macaca fascicularis*, six males, 10 females, and ages 4–5) anesthetized using a recommended concentration range of isoflurane. After careful scrutiny of the EEG traces, BS activity was detected in two monkeys (monkey O and monkey T) at 0.8–1.3% isoflurane during four separated experiments, out of which three experiments were performed on monkey O with a minimum separation of one and half month. The occurrence of BS was more frequent in monkey O than monkey T (Table 1). Further analysis on FC was based on all the data from monkey O and monkey T, including 20 runs with stable BS activity (BS group) and 8 runs with stable slow wave activity (SW group) from monkey O, 7 and 3 runs, respectively, from monkey T. Each run lasted 7 min with an inter-run interval of approximately 1 min within each experiment.

**Table 1:** Physiological information of all datasets from monkey O and monkey T.

Monkey ID	Session	Run	Group	Concentration of ISO (%)	BSR(%)	Heart Rate (bpm)	EtCO_2_ (mmHg)	Temperature (°C)
O	1	1	BS	1.2	10.9	141	28	36.6
O	1	2	BS	1.2	7.7	140	28	36.6
O	1	3	BS	1.2	12.8	136	29	36.8
O	1	4	BS	1.2	11.9	135	29	36.8
O	1	5	BS	1.2	2.6	135	29	36.9
O	1	6	BS	1.2	3.8	142	29	37
O	1	8	BS	1.2	1.2	135	28	37.1
O	1	9	BS	1.2	1.6	131	28	37.2
O	2	10	BS	1.3	38.9	131	26	37.1
O	2	11	BS	1.3	23.3	134	27	37
O	2	12	BS	1.3	32.1	136	27	37
O	2	13	BS	1.3	18	139	27	37
O	2	14	BS	1.3	27.9	140	27	37
O	2	15	BS	1.3	36.7	140	27	37
O	2	16	BS	1.3	26.3	142	28	37.1
O	2	17	BS	1.3	18.4	139	28	37
O	2	18	BS	1.1	14.1	140	28	37
O	2	19	BS	1.1	11.1	142	28	37
O	2	20	BS	1.1	16.5	140	28	37
O	3	27	BS	0.8	1.5	147	27	37.7

BS					15.87	138.25	27.8	36.995

O	3	7	SW	1.2	0	157	29	37
O	3	21	SW	0.8	0	147	26	37.3
O	3	22	SW	0.8	0	148	27	37.4
O	3	23	SW	0.8	0	149	27	37.5
O	3	24	SW	0.8	0	148	27	37.5
O	3	25	SW	0.8	0	149	27	37.7
O	3	26	SW	0.8	0	146	27	37.7
O	3	28	SW	0.8	0	147	27	37.8

SW						148.875	27.125	37.4875

*P*-value						<0.000	0.064	<0.000

T	4	31	BS	1.2	1.72	148	27	37.0
T	4	32	BS	1.2	0.56	156	27	37.1
T	4	33	BS	1.2	2.92	157	27	37.0
T	4	34	BS	1.2	1.68	160	27	36.8
T	4	35	BS	1.2	3.10	160	27	36.4
T	4	36	BS	1.2	0.86	159	26	36.3
T	4	38	BS	1.2	1.90	159	27	36.6

BS					1.82	157	26.857	36.743

T	4	29	SW	1.2	0	146	26	36.6
T	4	30	SW	1.2	0	148	26	36.8
T	4	37	SW	1.2	0	159	27	36.3

SW						151	26.333	36.567

*P*-value						0.125	0.120	0.421


### Animal Preparation

The animal preparation procedure was conducted in a similar manner to our previous work (Wang et al., 2013; Lv et al., 2016). Induction of anesthesia was achieved by intramuscular injection with ketamine (10 mg/kg, Gutian Pharma Co., Ltd., China) before data collection. After intubation, each monkey was ventilated with a mixture of isoflurane (2–2.5%, Lunan Pharma Co., Ltd., China) and pure oxygen via either a standard ventilator (CWE, Inc., Ardmore, PA, United States) in the preparation room or an MRI-compatible ventilator (CWE Inc., Weston, WI, United States) inside the scanner room. The monkey was maintained with intermittent positive-pressure ventilation to ensure a constant respiration rate (25–35 breaths/min). Vital signs including blood oxygenation, electrocardiogram (ECG), rectal temperature (Small Animal Instruments, Inc., Stony Brook, New York), respiration rate and end-tidal CO_2_ (Smiths Medical ASD Inc., Dublin, Ohio) were continuously monitored. Oxygen saturation was kept at over 95% and body temperature was kept constant using a heated water blanket (Gaymar Industries Inc., Orchard Park, New York). Lactated Ringer’s solution was given with a maximum rate of 10 ml/kg/hour during the anesthesia process (Logothetis et al., 1999).

To ensure adequate EEG data quality, we prepared the animal scalp by shaving and thorough cleaning with abrasive gel and alcohol swabs. A custom EEG cap made of stretchable material was fitted over the scalp and then fastened by a chinstrap. An ECG electrode was attached close to the heart to facilitate off-line removal of cardio-ballistic artifacts. To increase the signal-to-noise ratio, we injected conductive gel and ensured a low impedance (<5 kilo-ohms) at each electrode. The electrode filling holes were subsequently covered with medical tape to prevent the gel from drying out during recording. After setting up the EEG cap, the monkey was restrained within a water blanket in a sphinx-like position with the head protruding and facing forward. The animal’s head was secured using a custom-built MRI-compatible stereotaxic frame after local anesthetic (5% lidocaine cream) was applied around the ears to block peripheral nerve stimulation.

The isoflurane concentration was initially set to 1.2–1.3% within the recommended range in rs-fMRI research on isoflurane-anesthetized monkeys (Vincent et al., 2007; Hutchison et al., 2014), and adjusted by 0.05% increase or decrease to keep the continuously monitored physiological parameters within normal ranges (oxygen saturation: >95%; heart rate: 85–160 beats/min; temperature: 36–38°C; respiration rate: 22–35 breaths/min; end-tidal CO_2_: 24–30 mmHg) throughout the experiment. Datasets were collected at least 15 min after the adjustment of isoflurane level. Details of isoflurane concentration, BS ratio and physiological parameters for all included datasets were listed in Table 1.

### Simultaneous EEG-MRI Data Acquisition

MRI images were acquired at the Institute of Neuroscience on a 3T whole-body scanner (Trio; Siemens Healthcare, Erlangen, Germany) running with an enhanced gradient coil insert (AC88; 80 mT/m maximum gradient strength, 800 mT/m/s maximum slew rate) and a custom-built bird-cage volume coil with 8-channel array receiver. Whole-brain resting-state fMRI data were collected using a gradient echo planar sequence (TR = 2000 ms; TE = 29 ms; flip angle = 77°; slices = 32; matrix = 64 × 64; field of view = 96 mm × 96 mm; 1.5 mm × 1.5 mm in plane resolution; slice thickness = 2.5 mm; GRAPPA factor = 2). For each session, 5–10 runs were acquired and each run consisted of 200 functional volumes. A pair of gradient echo images (echo time: 4.22 and 6.68 ms) with the same orientation and resolution as EPI images were acquired to generate a field map for distortion correction of EPI images. High-resolution T1-weighted anatomical images were acquired using a MPRAGE sequence (TR = 2500 ms; TE = 3.12 ms; inversion time = 1100 ms; flip angle = 9°; acquisition voxel size = 0.5 mm × 0.5 mm × 0.5 mm; 144 sagittal slices). Six whole-brain anatomical volumes were acquired and further averaged for better brain segmentation and 3D cortical reconstruction.

Simultaneous EEG scalp recordings were acquired with BrainVision Recorder software using a BrainAmp MR amplifier and a 28-channel EEG cap customized for macaques (Brain Products GmbH, Gilching, Germany) with sintered Ag/AgCl ring electrodes. EEG signals from 21 active channels were sampled at 5000 Hz with a resolution of 0.5 μV per bit and measuring range of ±16 mV. The sampling clocks of the MR and EEG systems were synchronized using the SyncBox (Brain Products GmbH, Gilching, Germany), thus providing the time of fMRI volume acquisition for later gradient artifact removal in EEG signals.

### Data Analysis

#### MRI Data Preprocessing

Preprocessing of functional MRI images was conducted using the SPM8 toolbox^1^ and the FMRIB Software Library toolbox (FSL^2^). The first 10 volumes were discarded before preprocessing. The field map images of each session were applied to compensate for the geometric distortion of EPI images caused by magnetic field inhomogeneity using FSL FUGUE. After slice timing correction and motion correction, the corrected images were normalized to standard space of the monkey F99 atlas^3^ using an optimum 12-parameter affine transformation and nonlinear deformations, and then resampled to 1.5 mm cubic voxels and spatially smoothed with a 3 mm full-width at half-maximum (FWHM) isotropic Gaussian kernel. Linear drift of the volumes was removed, followed by regression of nuisance covariates (six head motion parameters, white matter and ventricle signals) and temporal filtering (0.01–0.1 Hz). To eliminate the BS coupling effect, we conducted another regression using BS pattern as an additional regressor.

#### EEG-MRI Coupling Analysis

We first assessed the coupling effect of BS pattern on filtered BOLD fluctuations. For each dataset with BS activity in EEG signal, the suppression episodes were automatically defined by thresholding the normalized amplitude summed across all available EEG channels. All labels of suppression onset or offset were visually inspected by an experienced anesthesiologist. The BS pattern was defined as a binary signal with zero indicating suppression epochs and one indicating non-suppression epochs. The time course of the binary BS pattern was then convoluted with the canonical hemodynamic response function (HRF) implemented in SPM8 as a regressor of interest. The significance of BS coupling effect on each voxel was evaluated via one-sample *t*-test. To account for the effect of repeated measure from the same subject, binary variables indicating whether the datasets were collected from a specific animal were included as covariates in the SPM model. Family-wise error (FWE) correction was applied to account for multiple comparisons with voxel-wise *P* < 0.001.

#### Whole-Brain Functional Connectivity Analysis

For the whole-brain FC analysis, we first parcellated the monkey brain into 82 cortical areas based on the Regional Map template (Kotter and Wanke, 2005; Bezgin et al., 2012) and 12 subcortical areas based on the INIA19 (Rohlfing et al., 2012) (see Supplementary Table S1 for a complete list of anatomical labels). Pearson’s correlation coefficients between the mean time courses of any pair of regions were calculated to represent their FC, resulting in a 94 × 94 connectivity network matrix for each dataset. Statistical significance of group differences were evaluated using linear regression analysis with binary variables of individuals as covariates in the GRETNA toolbox^4^. The edge-wise threshold of the significance level was set at *P* = 0.001, and cluster-level correction of *P* < 0.05 was applied to adjust the multiple comparison using the network-based statistic (Zalesky et al., 2010). Effect sizes measured via *Hedges’ g* values were calculated to evaluate the extent of group difference. In addition, we assessed the normalized distribution of disrupted edges within and between lobes. The bias caused by the unbalanced number of brain regions within different lobes was adjusted using the standardized residuals (*Z* scores), defined as the raw residuals (the difference between the observed edge count and expected edge count) divided by the square root of the expected edge count (Sheskin, 2003). The significance of the *Z* scores was estimated via non-parametric permutation (5,000 times). Specifically, the disrupted edges were randomly assigned across the whole brain network and the standardized residuals were re-calculated to generate the null distribution. The percentage of the assignments with a larger or equal *Z* score was defined as the *P* value. Bonferroni correction was applied for the correction of multiple comparisons.

#### Seed-Based Functional Connectivity Analysis

As the functional connections within the DMN and thalamocortical network are of particular interest during the anesthesia process, we further conducted seed-based functional network analysis. Bilateral posterior cingulate cortex (PCC) and bilateral thalamus defined in Regional Map parcellation were selected as seed regions, respectively. The averaged time course of fMRI signals within the seed region was then treated as a regressor of interest in the generalized regression model in the SPM toolbox. The connectivity strength between each voxel and the seed region was estimated via the beta coefficient in the regression model and statistically tested in each group using a one-sample *t*-test with covariates indicating individual animals. The group difference in voxel-wise connectivity was tested using a one-way repeated measure ANOVA. FWE correction was applied to account for multiple comparisons with voxel-wise *P* < 0.001.

## Results

### Brain-Wide BOLD Fluctuations Are Coupled With BS Activity

**FIGURE 1:**
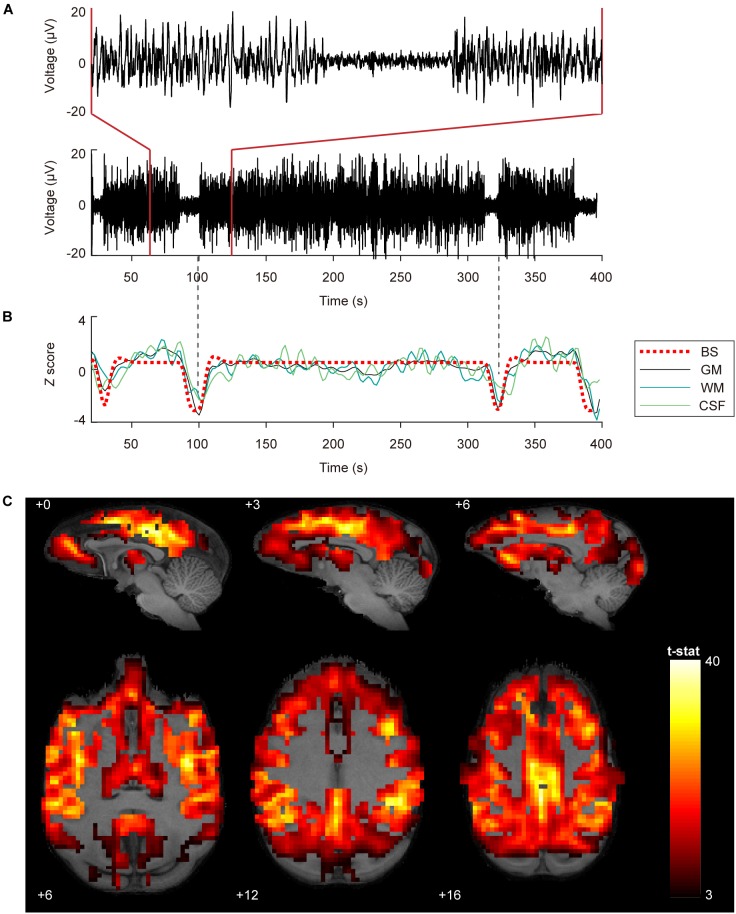
Coupling between BOLD fluctuations and burst suppression patterns in EEG signals. **(A)** EEG signals with burst suppression pattern simultaneously recorded during fMRI scanning from the central frontal electrode. The bottom and top panel show 380 and 60 s EEG, respectively. The time onset corresponds to the acquisition of the first MRI volume. **(B)** Burst suppression pattern with HRF convolution and averaged BOLD signals in gray matter (GM), white matter (WM), and cerebrospinal fluid (CSF). The dashed vertical lines in gray are aligned to the trough of the GM signal. **(C)** Group statistics (*n* = 27) shown as voxel-wise *t*-values of a generalized linear model modeling burst suppression pattern as a regressor of interest were displayed in bspmview (voxel-wise *P* < 0.001, FWE correction).

Figure 1 shows robust coupling between the BS pattern in EEG signals (Figure 1A) and the spontaneous fluctuations in BOLD signals after temporal filtering (Figure 1B, *Z* scored) in one typical dataset. Group analysis results indicated that the BOLD fluctuations of most neocortex were positively correlated with the EEG BS pattern (Figure 1C, voxel-wise *P* < 0.001, FWE correction), including the prefrontal cortex, temporal cortex, parietal cortex, somatosensory cortex, PCC, primary motor and premotor cortex, visural cortex, as well as thalamus.

### Altered Whole-Brain Functional Connectivity During BS Activity Compared to SW Activity

**FIGURE 2:**
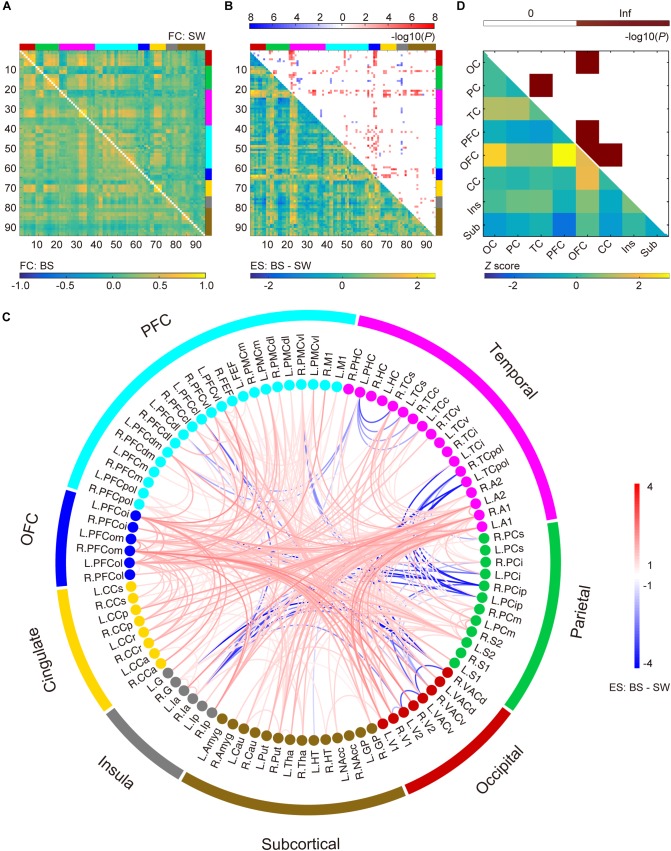
Altered functional connections during burst suppression activity compared to stable slow wave activity after the coupling effect with burst suppression pattern was corrected via regression. **(A)** Averaged functional connectivity (FC) matrices during burst suppression (BS, bottom-left) and slow wave (SW, top-right) activity. Covariates were regressed out before temporal filtering. **(B)** Effect sizes (ES, Hedges’ *g* value) of BS versus SW (bottom-left) and corresponding *P* values (top-right, *P* < 0.05, NBS correction with edge-wise *P* < 0.001). Brain nodes are organized according to the regions/lobes as listed in Supplementary Table S1. **(C)** Altered functional connections represented with node information. See Supplementary Table S1 for details of brain area abbreviations. **(D)** Normalized spatial distribution of disrupted connections across the brain (bottom-left) and the corresponding significance (top-right, *P* < 0.05, Bonferroni correction).

Results of whole-brain comparison based on datasets with or without removal of BS coupling effect were presented in Figure 2 and Supplementary Figure S3, respectively. Group-averaged connectivity networks from datasets with BS activity and stable SW activity are presented in Figure 2A. Group difference in each connection is represented using effect size (Hedges’ *g* value, bottom-left triangle in Figure 2B). A total of 371 functional connections showed significant differences between BS and SW groups (cluster-level corrected *P* < 0.00, NBS correction with edge-wise *P* < 0.001, top-right triangle in Figure 2B), including 317 increased and 54 decreased connections in BS group. As presented in Figure 2C, the BS group showed a substantial increase in cortical connectivity, accompanied by significant decrease mainly in connections between parietal and temporal/prefrontal cortex (PFC), and between insula and occipital/parietal cortex. The normalized distribution of altered connections across different brain lobes is summarized in Figure 2D (bottom-left triangle). The number of disrupted edges between certain lobes is significantly larger than the random distribution (*P* < 0.05, Bonferroni correction). The majority of within-lobe connections are concentrated in the orbitofrontal cortex (OFC). The majority of across-lobe connections are concentrated between OFC and prefrontal/cingulate/occipital cortex, and between parietal and temporal cortex.

### Increased Connectivity in Default Mode Network During BS Activity

**FIGURE 3:**
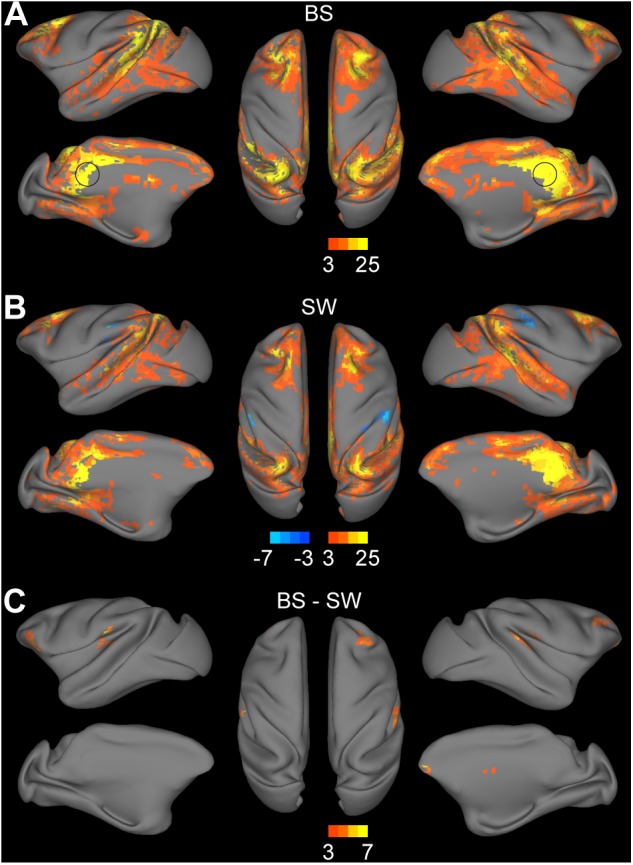
Altered functional connections across anesthesia levels in default mode network. **(A,B)** Voxel-wise connectivity with bilateral posterior cingulate cortex (indicated in black circle) in burst suppression (BS) and slow wave (SW) states. Volume metrics were mapped to cortical surface in CARET5. **(C)** Altered functional connections during BS activity compared to SW activity. All results were corrected for multiple comparisons at a significance level of *P* < 0.05 via FWE with voxel-wise *P* < 0.001.

The DMN was defined as the voxel-wise FC with bilateral PCC. The statistical results of the connectivity strength in BS and SW groups are presented in Figure 3A,B, respectively (voxel-wise *P* < 0.001, FWE correction). The BOLD activity in PCC was positively correlated with bilateral parietal cortex, medial and centrolateral prefrontal cortex, superior and ventral temporal cortex, primary motor and dorsolateral premotor cortex, anterior cingulate cortex and visual areas, and anticorrelated with secondary somatosensory and inferior parietal cortex during SW activity (Figure 3B). In comparison, there is an overall increase in DMN connectivity during BS activity (Figure 3A), particularly in the superior temporal cortex, prefrontal cortex, secondary somatosensory cortex and auditory cortex (Figure 3C, voxel-wise *P* < 0.001, FWE correction).

### Increased Thalamocortical Connections During BS Activity

**FIGURE 4:**
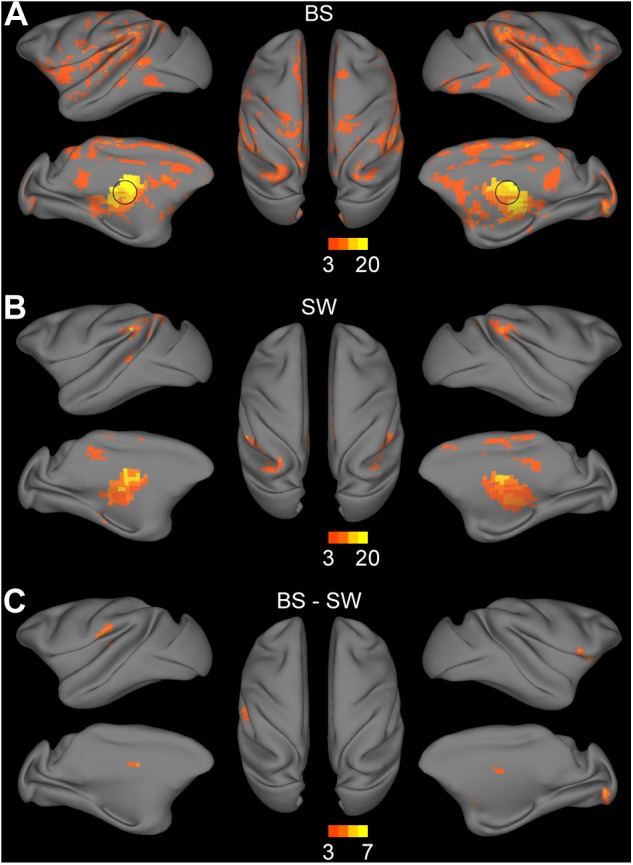
Altered functional connections across anesthesia levels in thalamocortical network. **(A,B)** Voxel-wise connectivity with bilateral thalamus (indicated in black circle) in burst suppression (BS) and slow wave (SW) states. Volume metrics were mapped to cortical surface in CARET5. **(C)** Altered functional connections during BS activity compared to SW activity. All results were corrected for multiple comparisons at a significance level of *P* < 0.05 via FWE with voxel-wise *P* < 0.001.

Thalamocortical connections were assessed by seed-based functional analysis with the bilateral thalamus as the seed region. Significant connections with the thalamus were observed in the bilateral striatum, medial prefrontal cortex, inferior parietal cortex, superior temporal cortex, auditory cortex, somatosensory cortex, posterior insula, visual cortex, anterior and posterior cingulate cortex during BS activity (Figure 4A, voxel-wise *P* < 0.001, FWE correction). Few thalamocortical connections to striatum, inferior parietal cortex, superior temporal cortex, retrosplenial and posterior cingulate cortex, somatosensory cortex and anterior visual area were observed during SW activity (Figure 4B, voxel-wise *P* < 0.001, FWE correction). The group comparison results further confirmed the overall increase during BS activity particularly in bilateral putamen, globus pallidus, right visual area, and left secondary somatosensory cortex (Figure 4B, voxel-wise *P* < 0.001, FWE correction).

## Discussion

### Robust BS Coupling Effect in the Raw BOLD Fluctuations

In the current study, we show dramatic fluctuations in BOLD signal co-occurring with EEG BS activity in the isoflurane-anesthetized monkey brain. The coupling between simultaneously recorded BOLD and EEG signals can be explained by the highly synchronized excitability of the entire cerebral cortex during BS activity. Convergent research has shown that BS is associated with a hyper-excited cortical state, where bursts can be evoked by sub-threshold stimulus or occur spontaneously (Steriade et al., 1994; Hartikainen et al., 1995; Hudetz and Imas, 2007; Kroeger and Amzica, 2007; Ferron et al., 2009). Electrophysiological studies indicate that neurons in almost the entire cerebral cortex exhibit a similar “on-off” pattern during this state, featuring stereotyped alteration between depolarizing events and electrical silence of the neuronal membrane (Steriade et al., 1994). The hyper-excited burst activity was positively correlated with the cerebral blood flow (CBF) fluctuations, which is the base of BOLD fluctuations, as indicated in a recent isoflurane-anesthetized rat research (Liu et al., 2011). Therefore, the BS coupling effect on BOLD fluctuations is supported by the physiological basis that synchronization of spontaneous high-voltage burst activity across a large population of neurons can result in the synchronization of hemodynamic fluctuations across different brain regions.

### Separating Specific and Non-specific Functional Connectivity During BS Activity

Although several lines of evidence suggest a coherent neural activity basis of resting-state FC, whether there is a specific FC signature that underlies the BS anesthesia depth remains unclear. As previously mentioned, neural activity that changes between two electrical phases with enormous disparity can lead to radical changes in BOLD fluctuations at a large time scale, which in turn results in an overwhelming increase in the correlation coefficients between whole-brain BOLD signals during BS activity (Supplementary Figure S3). This non-specific coupling effect of BS pattern is superimposed on the potential specific functional changes associated with anesthesia depth or consciousness loss (Figure 2), and obscures the latter effect.

In removing regular nuisance effect, we demonstrated that regression of WM and CSF signals before temporal filtering is more effective (Supplementary Figure S1). This is consistent with the previous findings in human rs-fMRI studies that nuisance-related variability such as head motion and cardiac artifacts can be better controlled when covariate regression is applied before temporal filtering rather than after filtering in the preprocessing procedure (Hallquist et al., 2013). Further evaluation of the BS coupling effect on preprocessed images indicated that BS related global effect as indicated in Figure 1C cannot be fully eliminated by using either preprocessing procedure (Supplementary Figure S2). Therefore, we added the HRF convoluted BS pattern as an additional regressor in the linear regression model to eliminate the global coupling effect in fMRI datasets with BS activity.

### Overall Increase in Functional Connectivity During BS Activity

An overall increase in functional connections during BS activity was observed compared to SW state in both whole-brain analysis (Figure 2) and seed-based analysis (Figure 3, 4). As non-specific increase in the correlation matrix due to BS pattern was well controlled in preprocessing, the remaining alterations across different anesthesia levels may largely reflect specific changes in a subgroup of networks or brain areas. Interestingly, most of the connectivity alterations took place within OFC, and between OFC and other cortical areas. The role of the OFC in BS activity or anesthesia progress merits further investigation. Reduced connectivity strength was observed in a subset of connections between insula and occipital-parietal cortex, suggesting the segregation of the limbic system from the neocortex along with the deepening of anesthesia level. Few alterations were observed within subcortical areas, although a significant increase from subcortical to temporal cortex, parietal cortex, OFC and PFC was detected, which was also verified in the seed-based analysis in thalamus.

### Preservation of DMN During BS Activity

The DMN is the most robust intrinsic network of the resting-state brain, which is associated with intrinsic processes such as mind wandering and self-reference (Buckner et al., 2008; Vanhaudenhuyse et al., 2011). Studies of alterations in DMN activity corresponding with different sleep stages indicate a potential correlation between the DMN and consciousness in humans (Raichle et al., 2001). Disconnection between the prefrontal cortex and the PCC has been observed during deep sleep (Horovitz et al., 2009; Samann et al., 2011). Subjects in sedation also exhibit a significant decrease in the default mode activity (Greicius et al., 2008; Martuzzi et al., 2010). Taken together, these findings suggest that the DMN may disappear at a certain stage during loss of consciousness. Nevertheless, preservation of the DMN connectivity under deep anesthesia was recently reported in both anesthetized monkeys and humans (Vincent et al., 2007; Boveroux et al., 2010; Hutchison and Everling, 2012). Our results displayed similar DMN patterns during both BS and SW activity as previous monkey studies (Vincent et al., 2007; Teichert et al., 2010). The robust findings of DMN under deep anesthesia indicate that the DMN may not be tightly associated with consciousness level (Buckner et al., 2008).

### Recovery of Thalamocortical Connectivity and the Role of Thalamus During BS Activity

Numerous studies have indicated the influence of anesthesia depth on thalamocortical connections. The presence of thalamocortical connectivity has been reported during light anesthesia where subjects just reached unconsciousness (Martuzzi et al., 2010; Mhuircheartaigh et al., 2010). Recent reports show diminished but detectable thalamocortical connectivity in anesthetized subjects associated with unconsciousness (White and Alkire, 2003; Boveroux et al., 2010; Liang et al., 2012). In the current study, few connections with striatum, cingulate cortex, primary sensory areas, inferior parietal cortex, and superior temporal cortex were observed during SW activity. In contrast, a substantial increase of connections to striatum, somatosensory, and visual cortex were detected during BS activity.

The thalamus is believed to be a crucial region for the synchronized burst activity of neocortex during the BS episodes of EEG (Brown et al., 2010). The first study of the neuronal mechanisms of BS (Steriade et al., 1994) confirmed a close correspondence between neocortical and thalamic activities through intracellular and multisite extracellular simultaneous recordings from cortex, thalamus, and upper brainstem. Despite the overwhelming predominance of cortical electrical silence during EEG suppression epochs, there were still rhythmic oscillations discharged to the cortex from a part of the thalamus. This thalamocortical oscillation was able to facilitate neuron firing or subthreshold depolarizing potentials as well as the revival of EEG activity. However, other studies indicated that cortical hyper-excitability during BS activity is favored by diminished cortical inhibition rather than thalamus induced excitation (Ferron et al., 2009). In our results, high synchronization between thalamic activity and BS pattern (Figure 1C) was revealed. Additionally, after the removal of BS coupling effect, there were significant connections from thalamus to primary sensory networks, which were weaker during SW activity. These enhanced thalamocortical connections might underlie the phenomenon that facilitated global burst activity could be induced by various kinds of subthreshold sensory stimuli under deep anesthesia with BS activity (Kroeger and Amzica, 2007). As for the role of thalamus in BS activity, further studies are desiderated.

### Anesthetic Sensitivity and the Importance of Simultaneous EEG Monitoring

For monitoring and defining anesthesia level, most fMRI studies generally employ two kinds of anesthesia depth monitoring indexes. One is behavioral endpoint, such as “loss of responsiveness” for human or “immobility” for animals. The other is end-tidal concentration or minimum alveolar concentration (MAC) for volatile anesthetics, both of which represent the anesthetic concentration level in subjects for inhibiting noxious stimulus (Eger et al., 1965). However, these indexes may not reflect the actual brain state (Antognini and Carstens, 2002; Ryu et al., 2018). The improved comprehension of EEG signals under anesthesia makes it a promising method suitable for more accurate control of anesthesia level (Freye and Levy, 2005; Purdon et al., 2013, 2015; Marchant et al., 2014).

Based on *post-hoc* analysis of the coherence of rs-fMRI correlation at various anesthesia levels, a limited range of isoflurane concentration (1.00–1.50%) is recommended to obtain stable FC (Hutchison et al., 2014). However, as mentioned previously, monkey fMRI studies with simultaneous EEG monitoring have recorded BS activity at 1.25–1.5% isoflurane (Vincent et al., 2007). In current work, we recorded BS activity in two out of 16 monkeys even at 0.8–1.3% isoflurane. The occurrence of BS activity at a commonly used concentration of isoflurane in fMRI studies suggests individual variance in anesthetic sensitivity at the brain level. Anesthetic sensitivity can be affected by both pharmacokinetic and pharmacodynamics factors and varies across different subjects (Chemali et al., 2015). Even in the same subject, there are many other confounding factors, including physiological status on the experiment day, and sleep quality before the experiment. The etiology and mechanism of anesthetic sensitivity in the brain merits further investigation.

Although BS activity induced by an isoflurane level as low as 0.8% rarely happens, it makes a strong argument for the potential occurrence of BS activity when delivering a widely-used range of concentrations in the field. Simultaneous EEG is necessary to monitor the actual brain state in regular rs-fMRI studies on anesthetized animals. More importantly, BS activity-induced changes in FC is robust. In current work, the occurrence of BS activity was spontaneous, resulting in inconsistent burst suppression ratio (BSR, range 0.56–38.9%). BSR is a commonly used parameter to describe the intensity of BS activity with larger BSR indicating higher probability of BS occurrence and deeper anesthesia level. In current study, robust increase of FC was observed across BS runs with different BSR. Greater enhancement was observed under a stable BS state with frequent and long-lasting suppression episodes than those with occasional occurrence of BS event. Further studies with deliberately controlled BSR are needed to investigate the relationship between BSR and BS effect on FC.

## Limitations

There are several limitations worth noting in this research. First, in consideration of the difficulty in controlling the occurrence of BS event and the individual variation in anesthetics sensitivity, present finding of the notably weak thalamocortical connectivity during SW activity could be affected by the relative small sample size. Second, another preliminary analysis on the current datasets suggested that the global synchronization strength during BS activity is potentially associated with BSR, which is not deliberately controlled in current study. Future studies with more subjects and precise control of BSR are needed to probe the biological underpinnings of the BOLD fluctuations at this specific anesthesia level. Third, to explore the influence of preprocessing procedures, we compared two conventional strategies used in monkey fMRI data analysis (Supplementary Figure S1). Other widely debated preprocessing steps such as global signal regression were not included. However, Scholvinck et al. (2010) showed that the often discarded global component of resting-state BOLD fluctuations is tightly coupled with underlying neural activity and may affect connectivity analysis. Fourth, there is a great chance that the global BS coupling effect is not fully excluded by regressing out the HRF convoluted BS pattern, although regression is the most commonly used approach to deal with nuisance variables with global influence. Lastly, pure oxygen was used in current experiments, which is considered as a probable confounding factor leading to more widespread connectivity in rs-fMRI studies (Nasrallah et al., 2015). However, we expect that the potential effect of hyperoxia is comparable in both BS and SW runs, as should impose very limited influence on the group difference in FC. Current results on specific alterations in FC during BS activity compared to SW activity should be interpreted with caution. However, the trend of overall increase in intrinsic FC during BS activity is robust and worth attention from researchers in the field.

## Conclusion

In summary, we present evidence showing a dramatic increase in inter-regional connectivity in fMRI network under deep anesthesia with BS activity. The enhanced connectivity can be explained by the coupling effect of BS pattern on whole-brain BOLD signals. This non-specific coupling effect can be well controlled by covariates regression conducted before temporal filtering with the HRF convoluted BS pattern as an additional regressor. After the coupling effect was separated from the connectivity matrix, we detected an overall increase in the functional connections under deep anesthesia with BS activity comparing to a lighter level with SW activity, including connections in DMN and thalamocortical networks. The orbitofrontal lobe are the most affected brain areas during BS activity. The non-linear changes in FC from light to deep anesthesia levels highlight the importance of future investigation on the physiological basis underlying BS activity. This is essential to clarify the mechanisms of anesthesia and coma states with BS events.

## Data Availability

The data that support the findings of this study are available on request from the corresponding author (ZW).

## Author Contributions

ZZ, ZheW, and YW designed research. ZZ, D-CC, and ZhiW performed research. D-CC and ZhiW analyzed the data. ZZ, D-CC, KZ, and ZheW wrote the manuscript.

## Conflict of Interest Statement

The authors declare that the research was conducted in the absence of any commercial or financial relationships that could be construed as a potential conflict of interest.

## Footnotes

1 http://www.fil.ion.ucl.ac.uk/spm

2 http://www.fmrib.ox.ac.uk

3 http://sumsdb.wustl.edu/sums/macaquemore.do

4 https://www.nitrc.org/projects/gretna
