# Supplementary material for: Isoflurane-Induced Burst Suppression Increases Intrinsic Functional Connectivity of the Monkey Brain
Source: Front Neurosci. 2019 Apr 11;13:296. doi: 10.3389/fnins.2019.00296 (PMC6470287; doi:10.3389/fnins.2019.00296)
Supplement: Supplementary file 1 [file Table_1.pdf]

Title: Isoflurane-induced burst suppression increases intrinsic functional connectivity of the monkey brain

Table S1. Cortical and subcortical parcellation and abbreviations

Figure S1. Effect of preprocessing procedures on functional connectivity in datasets with burst suppression activity

Figure S2. Residuals of BS coupling effect after image preprocessing with common strategy

Figure S3. Altered functional connections during burst suppression activity compared to stable slow wave activity without additional correction for coupling effect with burst suppression pattern

Table S1. Cortical and subcortical parcellation and abbreviations

Lobes	Number	Hemisphere	Number	Hemisphere	Abbreviation	Full name
Occipital	1	L	2	R	V1	Visual area 1 (primary visual cortex)
	3	L	4	R	V2	Visual area 2 (secondary visual cortex)
	5	L	6	R	VACv	Anterior visual area, ventral part
	7	L	8	R	VACd	Anterior visual area, dorsal part
Parietal	9	L	10	R	S1	Primary somatosensory cortex
	11	L	12	R	S2	Secondary somatosensory cortex
	13	L	14	R	PCm	Medial parietal cortex
	15	L	16	R	PCip	Intraparietal cortex
	17	L	18	R	PCi	Inferior parietal cortex
	19	L	20	R	PCs	Superior parietal cortex
Temporal	21	L	22	R	A1	Primary auditory cortex
	23	L	24	R	A2	Secondary auditory cortex
	25	L	26	R	TCpol	Temporal polar cortex
	27	L	28	R	TCi	Inferior temporal cortex
	29	L	30	R	TCv	Ventral temporal cortex
	31	L	32	R	TCc	Central temporal cortex
	33	L	34	R	TCs	Superior temporal cortex
	35	L	36	R	HC	Hippocampus
	37	L	38	R	PHC	Parahippocampal cortex
PFC	39	L	40	R	M1	Primary motor cortex
	41	L	42	R	PMCvl	Ventrolateral premotor cortex
	43	L	44	R	PMCdl	Dorsolateral premotor cortex
	45	L	46	R	PMCm	Medial premotor cortex
	47	L	48	R	FEF	Frontal eye field
	49	L	50	R	PFCvl	Ventrolateral prefrontal cortex
	51	L	52	R	PFCcl	Centrolateral prefrontal cortex
	53	L	54	R	PFCdl	Dorsolateral prefrontal cortex
	55	L	56	R	PFCdm	Dorsomedial prefrontal cortex
	57	L	58	R	PFCm	Medial prefrontal cortex
	59	L	60	R	PFCpol	Prefrontal polar cortex
OFC	61	L	62	R	PFCoi	Orbitoinferior prefrontal cortex
	63	L	64	R	PFCom	Orbitomedial prefrontal cortex
	65	L	66	R	PFCol	Orbitolateral prefrontal cortex
Cingulate	67	L	68	R	CCs	Subgenual cingulate cortex
	69	L	70	R	CCp	Posterior cingulate cortex
	71	L	72	R	CCr	Retrosplenial cingulate cortex
	73	L	74	R	CCa	Anterior cingulate cortex
Insula	75	L	76	R	G	Gustatory cortex
	77	L	78	R	Ia	Anterior insula
	79	L	80	R	Ip	Posterior insula
Subcortical	81	L	82	R	Amyg	Amygdala
	83	L	84	R	Cau	Caudate
	85	L	86	R	Put	Putamen
	87	L	88	R	Tha	Thalamus
	89	L	90	R	HT	Hypothalamus
	91	L	92	R	NAcc	Nucleus accumbens
	93	L	94	R	GP	Globus pallidus

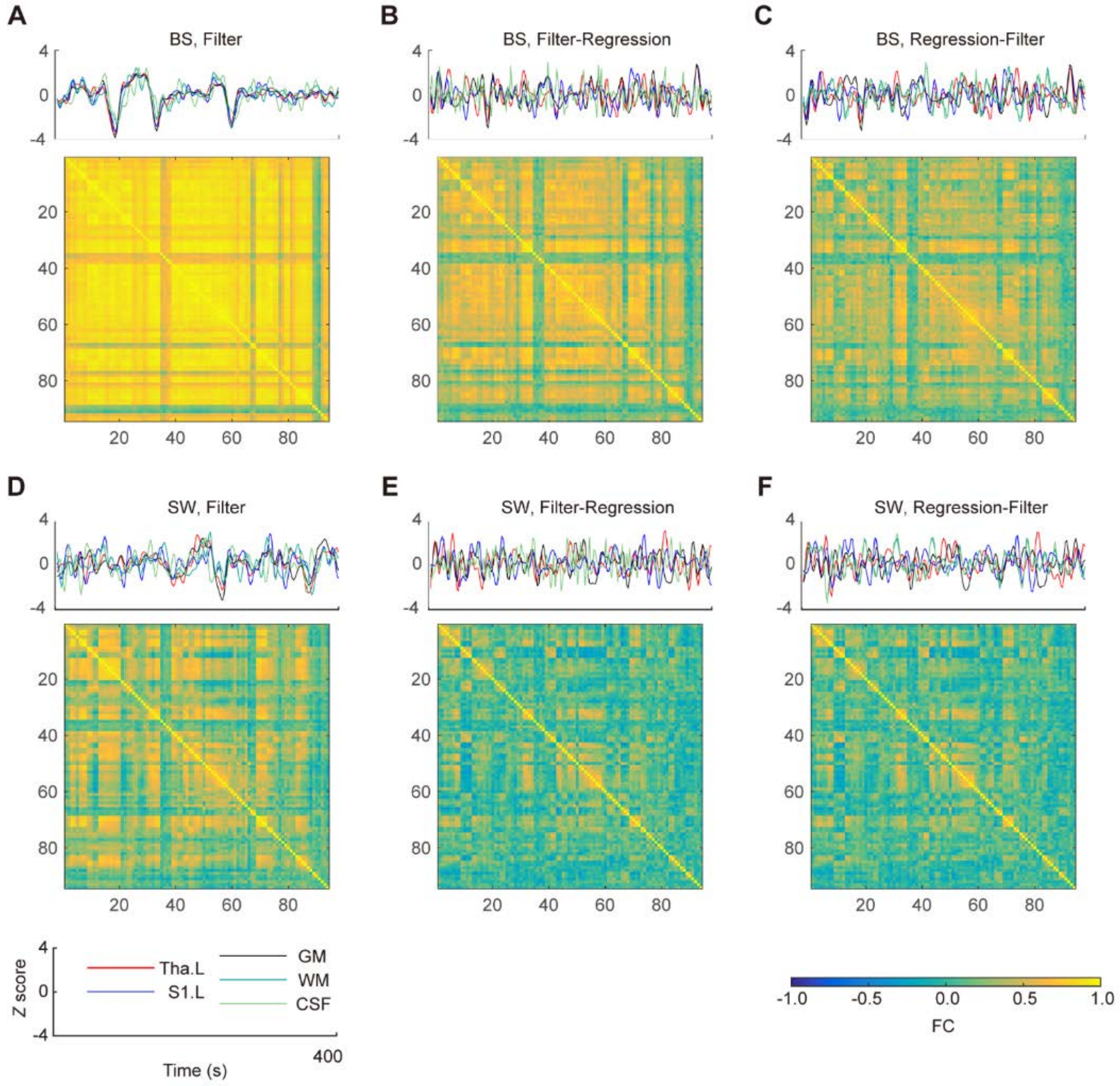

Figure S1. Effect of preprocessing procedures on functional connectivity in datasets with burst suppression activity. **(A-C)** Functional connectivity between 94 brain regions of a single dataset with burst suppression activity. Different preprocessing procedures were applied to the BOLD signals: **(A)** band-pass filtering at 0.01 - 0.1 Hz; **(B)** covariates regression (motion parameters, averaged WM signal, and averaged CSF signal) after filtering; **(C)** covariates regression before filtering. Averaged BOLD signals in left thalamus (Tha.L), left primary somatosensory cortex (S1.L), gray matter (GM), white matter (WM) and cerebrospinal fluid (CSF) are presented in upper panel. **(D-F)** Functional connectivity of a single dataset with stable slow wave activity. The data was collected from the same experiment as in **(A-C)** with the same inhaled concentration of isoflurane (1.20%). BS, burst-suppression; SW, slow wave; FC, functional connectivity.

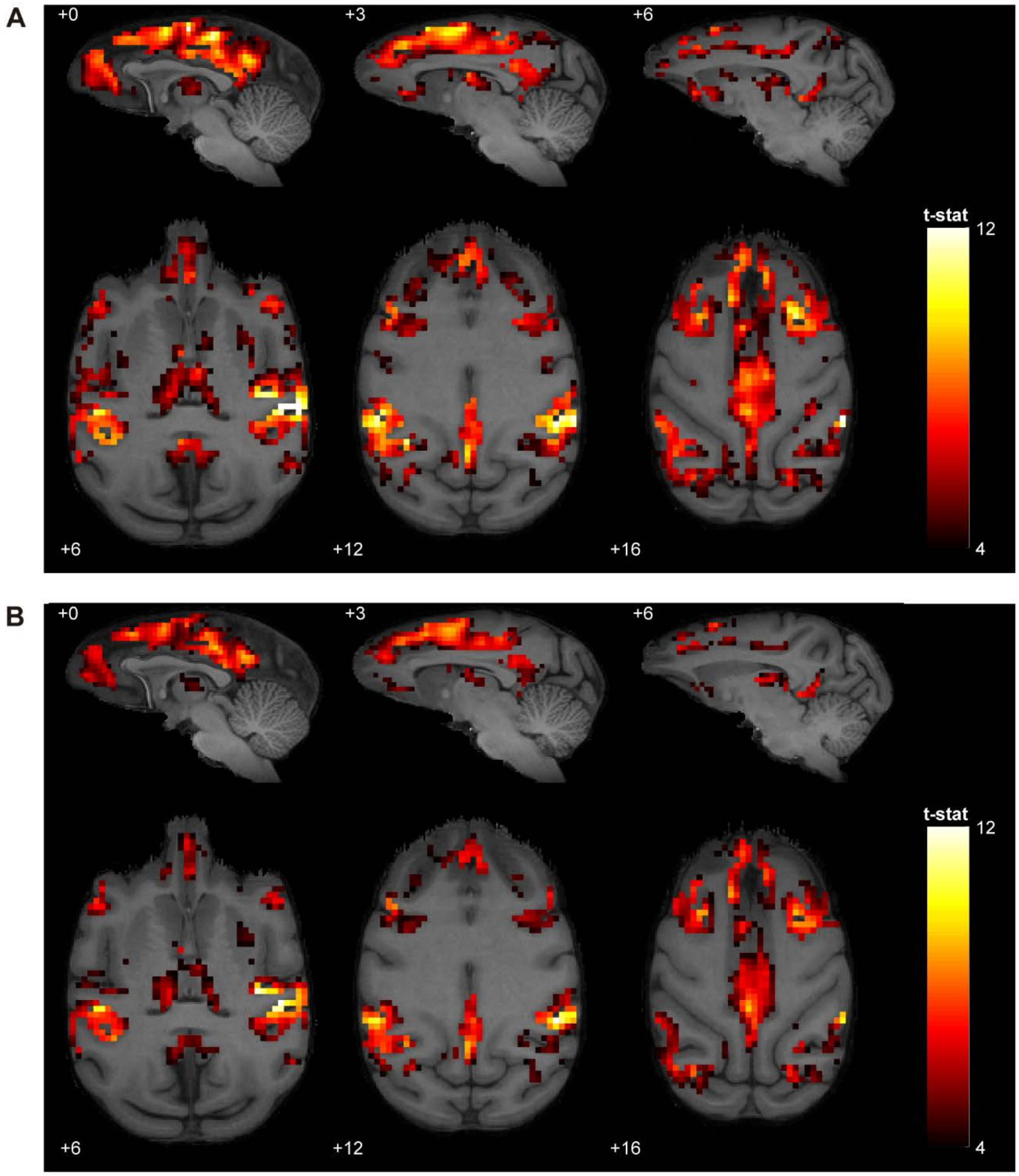

Figure S2. Residuals of BS coupling effect after image preprocessing with common strategy. **(A-B)** Same analysis as in **Figure 1C** was applied on the data with covariates regression conducted after filtering **(A)** or before filtering **(B)**. Group statistics (*n* = 27) shown as voxel-wise t-values of a generalized linear model modeling burst suppression pattern as a regressor of interest were displayed in bspmview (voxel-wise $P < 0.001$, FWE correction).

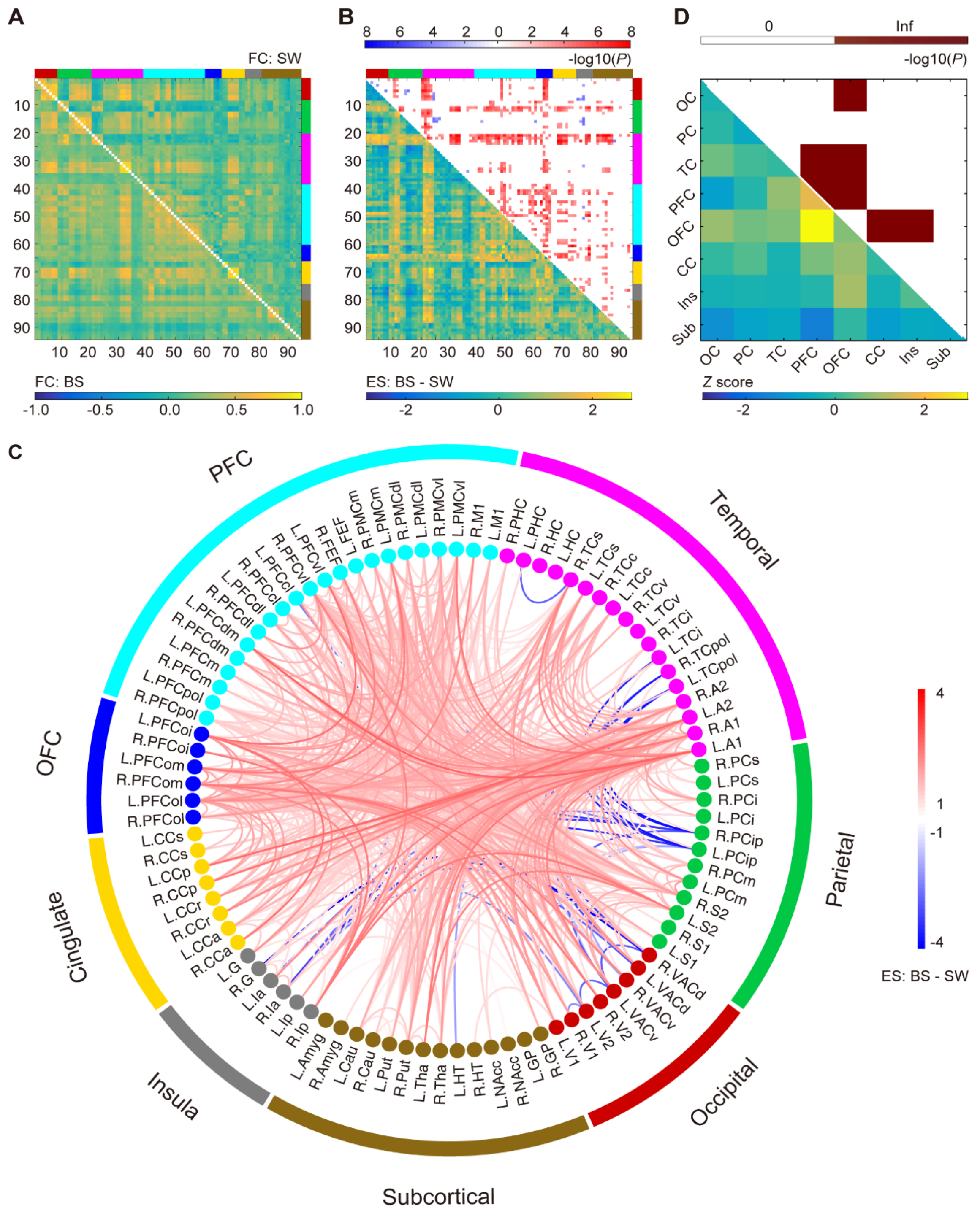

Figure S3. Altered functional connections during burst suppression activity compared to stable slow wave activity without additional correction for coupling effect with burst suppression pattern. **(A)** Averaged functional connectivity (FC) matrices during burst suppression (BS, bottom-left) and slow wave (SW, top-right) activity. Covariates were regressed out before temporal filtering. **(B)** Effect sizes (ES, Hedges' *g* value) of BS versus SW (bottom-left) and corresponding *P* values (top-right, $P < 0.05$, NBS correction with edge-wise $P < 0.001$). Brain nodes are organized according to the regions/lobes as listed in Table S1. **(C)** Altered functional connections represented with node information. See Table S1 for details of brain area abbreviations. **(D)** Normalized spatial distribution of disrupted connections across the brain (bottom-left) and the corresponding significance (top-right, $P < 0.05$, Bonferroni correction).
